# DEAD-box RNA helicase 10 inhibits porcine circovirus type 3 replication by interacting with the viral capsid protein and activating interferon responses

**DOI:** 10.1128/jvi.00576-25

**Published:** 2025-05-09

**Authors:** Jianwei Zhou, Ning Zhu, Qianhong Dai, Haoyu Sun, Jie Zhao, Yonghui Qiu, Beiyi Zhou, Dedong Wang, Yongqiu Cui, Jinshuo Guo, Xufei Feng, Lei Hou, Jue Liu

**Affiliations:** 1 College of Veterinary Medicine, Yangzhou University 614704, Yangzhou, China; 2 Jiangsu Co-innovation Center for Prevention and Control of Important Animal Infectious Diseases and Zoonoses, Yangzhou University 38043, Yangzhou, China; College of Agriculture & Life Sciences, University of Arizona, Tucson, Arizona, USA

**Keywords:** porcine circovirus type 3, capsid protein, DEAD-box RNA helicase 10, antiviral activity, viral replication

## Abstract

Porcine circovirus type 3 (PCV3) is an emerging pathogen that causes porcine dermatitis and nephropathy syndrome-like symptoms, multisystemic inflammation, and reproductive failure. The PCV3 capsid (Cap) protein interacts with DEAD-box RNA helicase 10 (DDX10), a protein that functions primarily through regulating interferon (IFN)-β production to exert its antiviral activity. However, how the interaction between DDX10 and PCV3 Cap regulates viral replication remains unknown. We used Western blotting, interaction assays, and knockdown analyses to observe impaired PCV3 proliferation in transiently DDX10-overexpressing cells, as indicated by decreased viral protein expression levels and virus production. In contrast, PCV3 replication increased upon small interfering RNA-mediated *DDX10* depletion. Furthermore, DDX10 positively regulated IFN-β production and interferon-stimulated gene expression, inhibiting PCV3 replication. Mechanistically, PCV3 Cap co-localized and interacted with DDX10, and the N-terminal nuclear localization signal of PCV3 Cap and the helicase domain of DDX10 were essential for the Cap-DDX10 interaction. Furthermore, PCV3 infection decreases DDX10 expression to antagonize its antiviral activity. These results show that DDX10 antagonizes PCV3 replication by interacting with the PCV3 Cap protein and activating IFN-β responses, which provides important insight into the prevention and control of PCV3 infection.

**IMPORTANCE:**

Clarifying how host factors contribute to infection with PCV3, a newly discovered pathogen associated with multiple clinicopathological signs in swine, helps elucidate viral pathogenesis. The PCV3 Cap protein has been shown to interact with DDX10, a crucial protein that regulates RNA virus replication. Herein, we further demonstrated that DDX10 expression is downregulated in PCV3-infected cells and antagonizes the replication of PCV3 and that DDX10 increases interferon-β and interferon-stimulated gene levels to inhibit PCV3 replication by binding to the PCV3 Cap. In addition, PCV3 infection decreases DDX10 expression to antagonize its antiviral activity. These results reveal a molecular mechanism by which DDX10 antagonizes PCV3 replication by binding to the PCV3 Cap protein and activating IFN signals, thereby providing important targets for preventing and controlling PCV3 infection.

## INTRODUCTION

Porcine circovirus type 3 (PCV3) is a recently identified porcine circovirus in sows with porcine dermatitis and nephropathy syndrome (PDNS), multisystemic inflammation, and reproductive failure in the United States (1, 2). PCV3 infection subsequently spread worldwide, resulting in substantial economic losses (3–5). There are nearly 50 years of PCV3 infection (6–8). In 2019, PDNS reemerged in piglets infected with PCV3, implying that PCV3 is pathogenic and induces PDNS-related clinical symptoms (9–12). Many studies have clarified how cellular factors interacting with the PCV3 capsid (Cap) protein contribute to its infection (2, 9, 10, 12–14). For example, PCV3 enters PK-15 cells via clathrin- and dynamin-2-mediated endocytosis (15). Nucleolar nucleophosmin-1 promotes virus replication by binding to the PCV3 capsid (Cap) protein (16, 17). Heme oxygenase-1, carbon monoxide, and biliverdin inhibit PCV3 replication (18). However, the function of the PCV3 Cap protein in the viral replication cycle has only been partially demonstrated (19–22).

The PCV3 Cap protein has been found to interact with DEAD-box RNA helicase 10 (DDX10) (23), which shares a highly conserved DEAD motif and is attributed to RNA helicase superfamily II (24). Almost 40 DExD/H-box helicases (DDXs) that bear conserved motifs (Q, I, Ia, Ib, Ic, II, III, IV, IVa, V, Va, Vb, and VI) involved in RNA binding, decay, ATP binding, and hydrolysis have been characterized (25, 26). However, in addition to regulating RNA metabolism, DDXs are related to viral infection by binding to virus-encoded proteins and modulating antiviral innate immunity (27–29). For example, DDX3X increases interferon (IFN)-β production via interferon regulatory factor 3 (IRF3) and the nuclear factor kappa B signaling axis to prohibit dengue virus proliferation (30). DDX60 orchestrates RIG-I signaling and increases IFN-I levels to inhibit hepatitis C and vesicular stomatitis virus replication (31). Furthermore, the binding of DDX17 to zinc finger CCCH-type containing antiviral 1 induces the decay of human immunodeficiency virus mRNA to suppress virus proliferation (32, 33). Porcine reproductive and respiratory syndrome virus infection induces DDX5 redistribution from the nucleus to the cytoplasm to bind to the viral nsp9 protein, facilitating virus proliferation (34). The ternary complexes DDX1, DDX21, and DHX36 govern host innate immunity, and deprivation of any compound component represses cellular immune responses, and caspase-dependent DDX21 cleavage disrupts cellular antiviral immune responses (35–37). Porcine reproductive and respiratory syndrome virus induces DDX10 degradation via SQSTM1-dependent selective autophagy to disrupt antiviral immune responses (38). In addition, RIG-I, DDX3X, DDX41, and DDX60 increase IFN-β expression to suppress virus proliferation (39–42). Although DDX10 interacts with PCV3 Cap, whether this binding modulates virus replication is still unclear.

In this study, we demonstrate that DDX10 expression inhibits PCV3 replication. We further show that DDX10 increased IFN-β levels and interferon-stimulated gene (ISG) expression, prohibiting PCV3 replication. PCV3 infection downregulated DDX10 to antagonize its antiviral activity. Mechanistically, PCV3 Cap co-localized and interacted with DDX10, and the PCV3 Cap nuclear localization signal (NLS) and helicase domain of DDX10 were essential for the Cap-DDX10 interaction. These results provide novel insight into the prevention and control of PCV3 infection.

## RESULTS

### PCV3 infection reduces the expression of the RNA helicase DDX10

**Fig 1:**
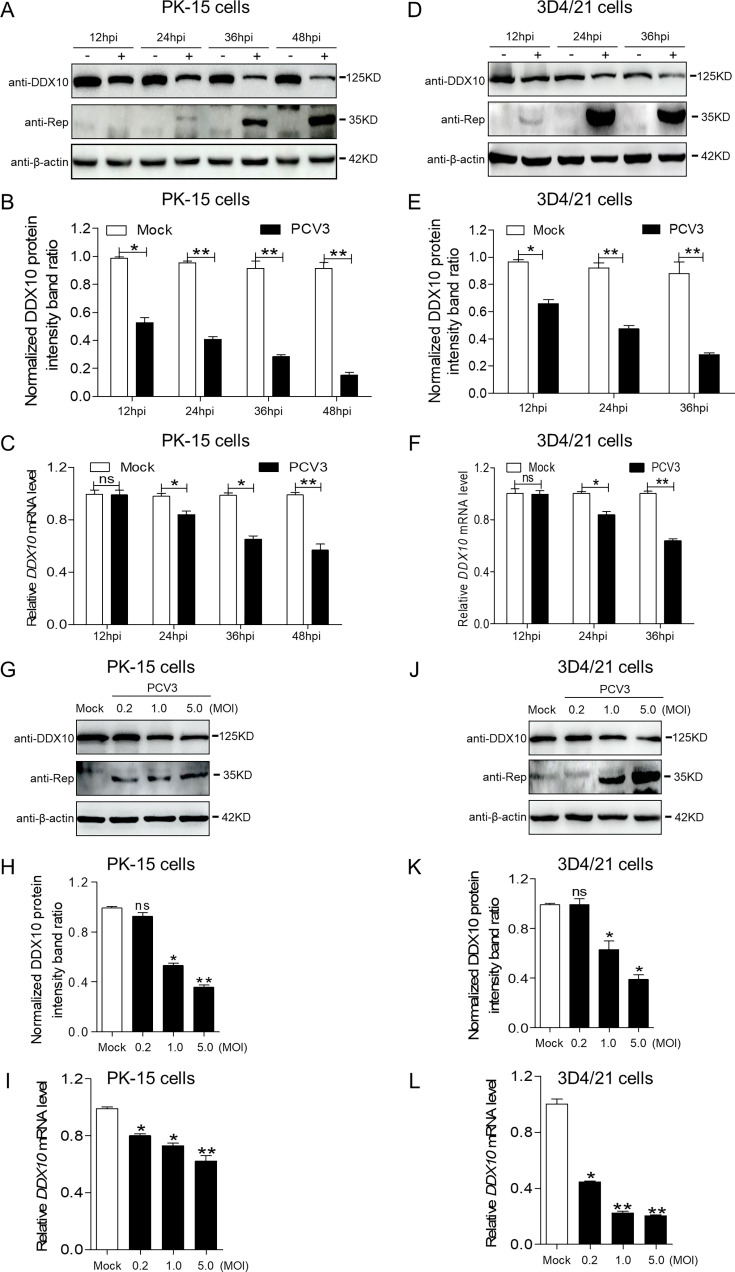
PCV3 infection reduces DDX10 expression in PK-15 and 3D4/21 cells. (**A**) PK-15 cells were mock-infected or infected with PCV3 at an MOI of 1 for 12, 24, 36, and 48 h, and whole-cell lysates were collected and then analyzed by immunoblotting with anti-DDX10, anti-replicase (Rep), and anti-β-actin antibodies, respectively. (**C**) Real-time quantitative PCR measurements of the *DDX10* mRNA abundance at the indicated times described in panel **A**. (**D and F**) Proteins and *DDX10* mRNA were extracted from PCV3-infected or mock-infected 3D4/21 cells at 12, 24, and 36 hpi and were analyzed by immunoblotting with anti-DDX10, anti-Rep, and anti-β-actin antibodies and RT-qPCR (**D**), respectively. (**G and J**) PK-15 and 3D4/21 cells were mock-infected or infected with PCV3 at the indicated MOI of 0.2, 1.0, and 5.0 for 24 h, and whole-cell lysates were collected and then analyzed by immunoblotting with anti-DDX10, anti-Rep, and anti-β-actin antibodies, respectively. (**I and L**) RT-qPCR measurements of the *DDX10* mRNA abundance in PK-15 and 3D4/21 cells at the indicated MOI described in panels **G and J**, respectively. ssion was normalized to the β-actin or *GAPDH* mRNA level. (**B, E, H, and K**) The protein intensity band ratio of DDX10 to β-actin was normalized to control conditions in panels **A, D, G, and J**, respectively. Data are presented as means ± SD of three independent biological experiments. ns, not significant. *P < 0.05, **P < 0.01.

DDX10 has been shown to interact with the PCV3 Cap protein (23). To determine the expression of DDX10 during PCV3 infection, we analyzed the kinetics of endogenous DDX10 expression in different cell types infected with PCV3 at a multiplicity of infection (MOI) of 1 via Western blotting and quantitative real-time reverse transcription PCR. The protein levels of DDX10 in PCV3-infected PK-15 cells were significantly decreased at 12, 24, 36, and 48 h post-infection (hpi) (Fig. 1A), which was further confirmed by an analysis of the relative densitometric ratios of DDX10 and β-actin (the corresponding internal reference) (Fig. 1B, *P* < 0.05). The mRNA level of *DDX10* in PCV3-infected PK-15 cells also significantly decreased at the indicated time points (Fig. 1C, *P* < 0.05). Similar results were observed in PCV3-infected 3D4/21 cells at 12, 24, and 36 h post-infection (Fig. 1D through F, *P* < 0.05), demonstrating that DDX10 expression was downregulated when the cells were infected with PCV3. This downregulation occurred in a dose-dependent manner in PCV3-infected PK-15 cells (Fig. 1G through I, *P* < 0.05) or 3D4/21 cells (Fig. 1J through L, *P* < 0.05) at MOIs of 0.2, 1.0, and 5.0. These results showed that PCV3 infection reduced the protein and transcriptional levels of the RNA helicase DDX10.

### DDX10 expression inhibits PCV3 replication

**Fig 2:**
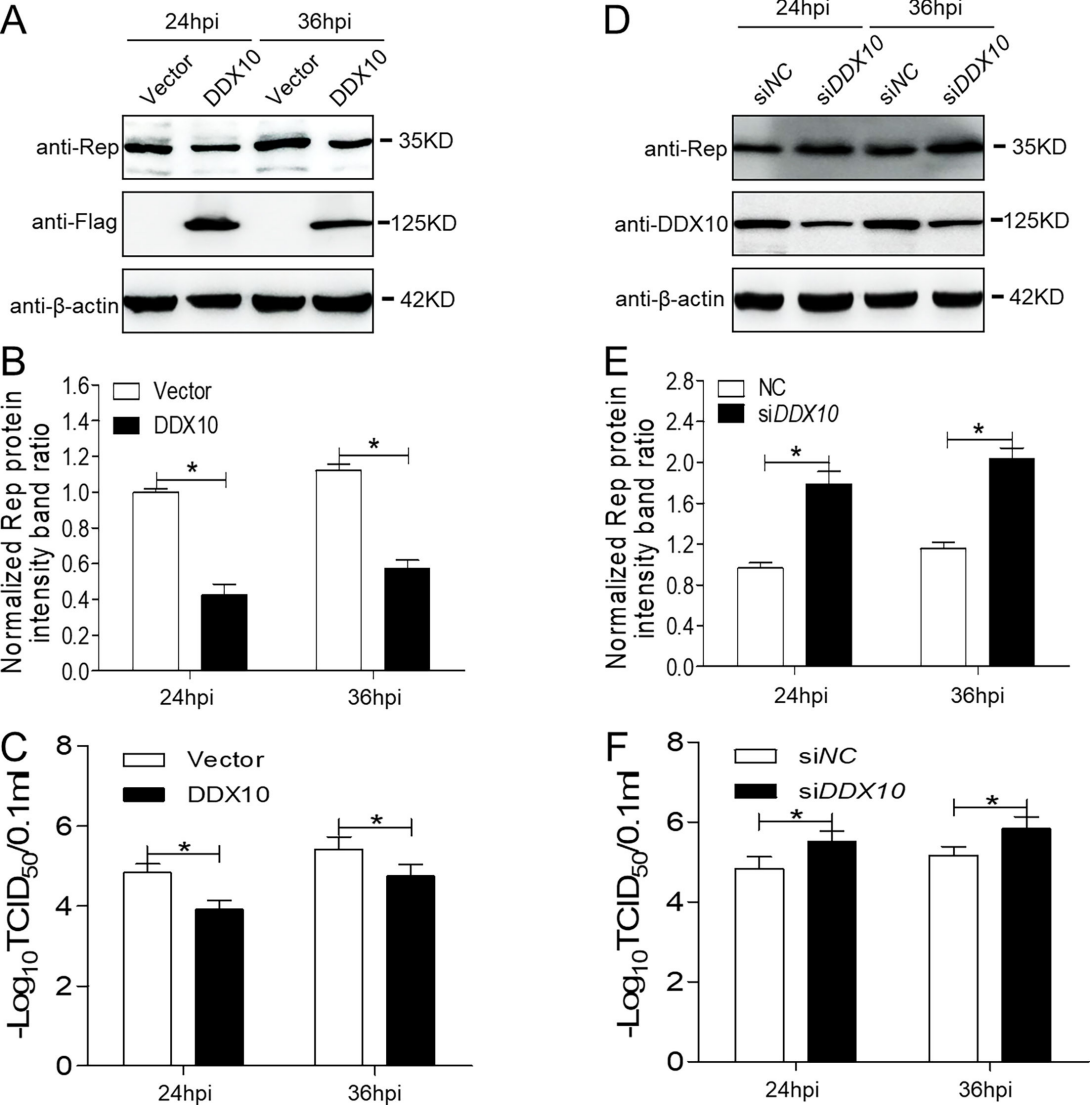
DDX10 inhibits the replication of PCV3. (**A and D**) Immunoblotting of the proteins Rep, DDX10, FLAG, and β-actin in DDX10-overexpressing or *DDX10*-silenced PK-15 cells. The cells were infected with PCV3 at an MOI of 1 for the indicated time, and siCON-transfected or empty vector-transfected cells served as negative controls. (**C and F**) Fifty percent tissue culture infective dose (TCID_50_) values of PCV3 in samples from panels **A** and **D**. DDX10-overexpressing or *DDX10*-silenced PK-15 cells were infected with PCV3 at an MOI of 1. Viral stocks were harvested at 24 and 36 hpi. (**B and E**) The protein intensity band ratio of Rep to β-actin was normalized to control conditions in panels **A and D**, respectively. Data are presented as mean ± SD from three independent biological experiments. *P < 0.05.

To investigate the role of DDX10 during PCV3 infection, DDX10-overexpressing cells were infected with PCV3 at an MOI of 1, and viral replication was evaluated by determining the replicase (Rep) protein expression levels and the virus titer. Rep protein expression was significantly lower in DDX10-overexpressing cells than in PCV3-infected empty vector cells (Fig. 2A and B, *P* < 0.05). Furthermore, the replicative ability of PCV3 in DDX10-overexpressing cells was approximately 10-fold lower than that in control cells during infection (Fig. 2C, *P* < 0.05). These results suggested that PCV3 replication was downregulated in DDX10-overexpressing cells.

Since DDX10 overexpression inhibits PCV3 replication, we aimed to determine whether silencing DDX10 expression promotes PCV3 replication. A small interfering RNA (siRNA) specific for *DDX10* (si*DDX10*) was used to reduce the DDX10 protein level. *DDX10*-silenced cells were infected with PCV3 at an MOI of 1. PCV3 Rep expression levels were significantly increased in *DDX10*-silenced cells than in control cells during infection (Fig. 2D and E, *P* < 0.05). Similarly, PCV3 viral progeny production was significantly increased in *DDX10*-silenced cells (Fig. 2F, *P* < 0.05), suggesting that silencing *DDX10* promotes PCV3 replication. These results indicated that DDX10 inhibited PCV3 replication.

### PCV3 Cap co-localizes and interacts directly with DDX10

**Fig 3:**
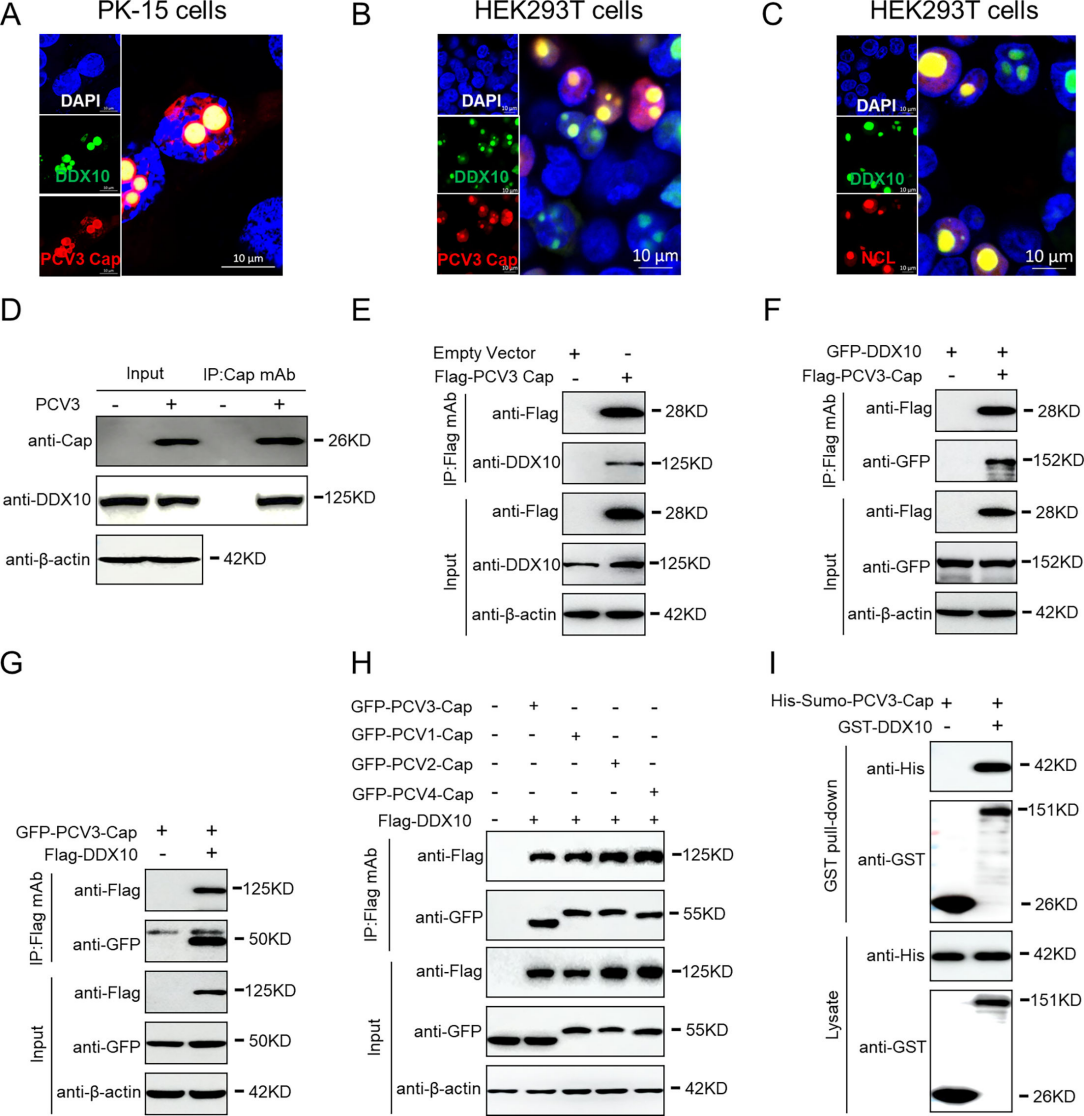
PCV3 Cap co-localizes and directly interacts with DDX10. (**A**) The co-localization of the viral protein Cap with DDX10. PK-15 cells were co-transfected with GFP-DDX10 and mCherry-PCV3-Cap for 24 h. (**B and C**) The co-localization of the viral protein DDX10 with PCV3 Cap or NCL. HEK293T cells were co-transfected with GFP-DDX10 and mCherry-PCV3-Cap (**B**) or GFP-DDX10 with mCherry-NCL (**C**) for 24 h. Cells were fixed and stained with 4′,6′-diamidino-2-phenylindole and then observed under a confocal microscope. (**D and E**) Immunoprecipitation analysis. PK-15 cells were infected with PCV3 at an MOI of 1 for 24 h or transfected with an empty vector and FLAG-PCV3-Cap for 48 h. The cell lysates were immunoprecipitated with anti-Cap mAbs or FLAG beads, followed by immunoblotting with anti-Cap, anti-FLAG, anti-DDX10, and anti-β-actin antibodies. (**F and G**) Co-immunoprecipitation analysis. HEK293T cells were co-transfected with FLAG-PCV3-Cap and GFP-DDX10 or FLAG-DDX10 and GFP-PCV3-Cap for 48 h, and then the cell lysates were immunoprecipitated with FLAG beads and analyzed by immunoblotting with anti-FLAG, anti-GFP, and anti-β-actin antibodies. (**H**) Co-immunoprecipitation analysis. HEK293T cells were co-transfected with FLAG-DDX10 and GFP-PCV3-Cap, GFP-PCV1-Cap, GFP-PCV2-Cap, or GFP-PCV4-Cap for 48 h. The cell lysates were then immunoprecipitated with FLAG beads and analyzed by immunoblotting with anti-FLAG, anti-GFP, and anti-β-actin antibodies. (**I**) A total of 500 ng of purified His-Sumo-PCV3-Cap separately mixed with the equal amount of purified glutathione *S*-transferase (GST). GST-DDX10 proteins were pulled down with GST beads and then subjected to GST pull-down assays and immunoblotted with corresponding antibodies.

We observed the intracellular distributions of DDX10 and PCV3 Cap via confocal microscopy to further investigate the relationship between PCV3 Cap and DDX10 during transfection. DDX10 co-localized with PCV3 Cap or nucleolin (NCL) in PK-15 cells co-transfected with GFP-DDX10 and mCherry-PCV3-Cap (Fig. 3A), in HEK293T cells with GFP-DDX10 and mCherry-PCV3-Cap (Fig. 3B), or in HEK293T cells with GFP-DDX10 and mCherry-NCL (Fig. 3C). NCL is a nucleolus-resident protein (43), and DDX10 overlaps with PCV3 Cap or NCL. Immunofluorescence assays revealed that PCV3 Cap or DDX10 is also a nucleolus-located protein that exhibits nucleolar localization. To investigate the endogenous interaction between PCV3 Cap and DDX10, PK-15 cell lysates infected with PCV3 or transfected with FLAG-PCV3-Cap were immunoprecipitated with anti-Cap monoclonal antibodies (mAbs) or FLAG beads and probed for the presence of DDX10 with an anti-DDX10 polyclonal antibody. The results showed that PCV3 Cap interacted with the endogenous DDX10 protein (Fig. 3D and E). To further verify the interaction between DDX10 and PCV3 Cap, HEK293T cells were co-transfected with FLAG-PCV3-Cap and GFP-DDX10 or GFP-PCV3-Cap and FLAG-DDX10 and then immunoprecipitated with FLAG beads. DDX10 interacted with PCV3 Cap (Fig. 3F and G). To investigate whether the Cap protein of PCV1, PCV2, and PCV4 also interacts with DDX10, HEK293T cells co-transfected with FLAG-DDX10 and GFP-PCV3-Cap, GFP-PCV1-Cap, GFP-PCV2-Cap, or GFP-PCV4-Cap were immunoprecipitated with FLAG beads. The results indicated that the PCV1, PCV2, and PCV4 Cap proteins also interacted with DDX10 (Fig. 3H). Additionally, glutathione-*S*-transferase (GST) pull-down assays were performed to investigate whether PCV3 Cap interacts directly with DDX10. GST pull-down experiments were performed using purified His-Sumo-PCV3-Cap and GST-DDX10 or GST control. The results revealed that the GST-DDX10 protein interacted directly with His-Sumo-PCV3-Cap (Fig. 3I). Collectively, these results demonstrate that PCV3 Cap co-localizes and binds directly to DDX10.

### The NLS of PCV3 Cap is crucial for interaction with DDX10

**Fig 4:**
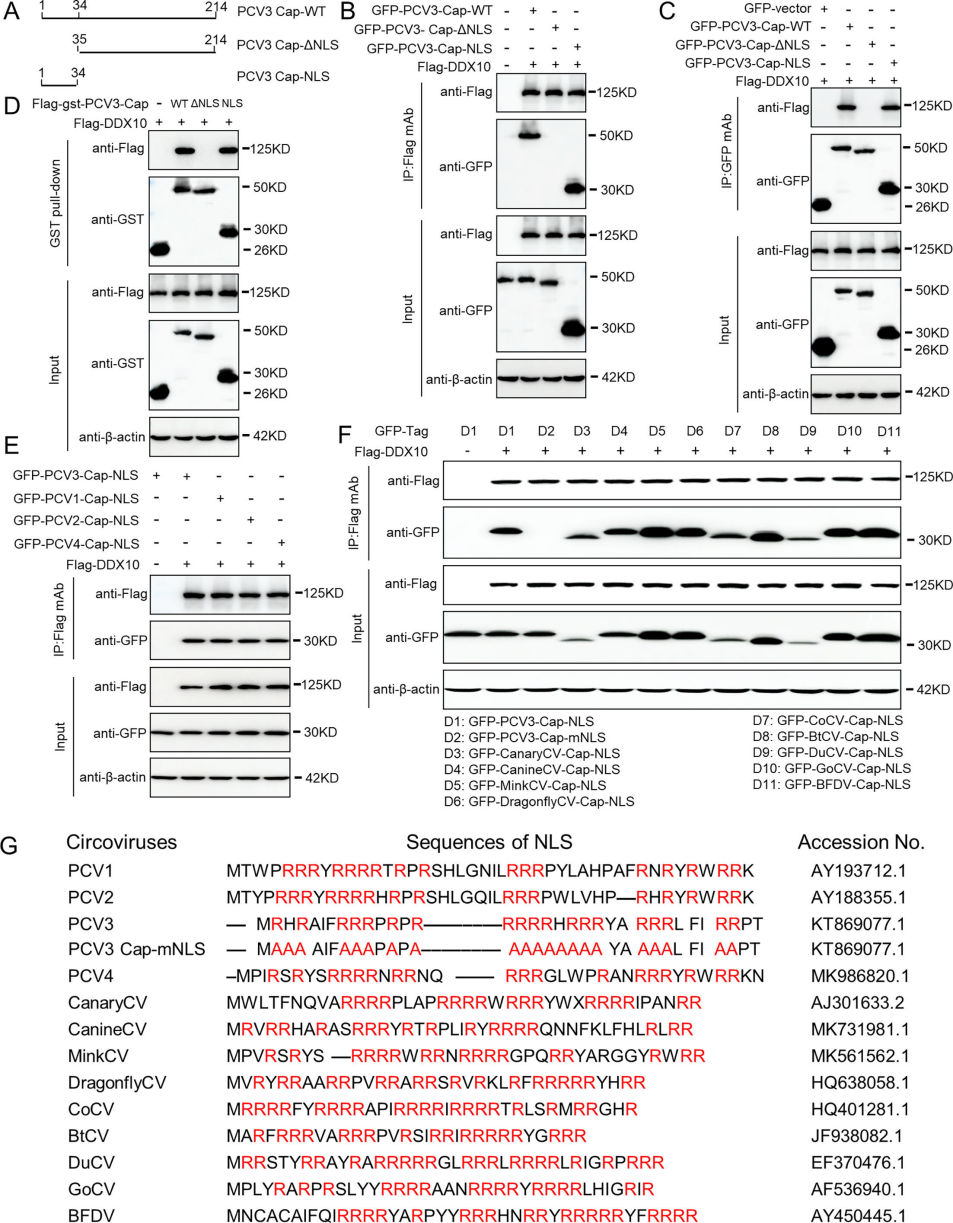
Binding domain identification of PCV3 Cap with DDX10. (**A**) Schematic representation of the truncation mutants of the PCV3 Cap used in this study. (B–D) The NLS of PCV3 Cap interacted with DDX10. HEK293T cells were co-transfected with plasmids encoding full-length PCV3 Cap or truncation mutants fused with a GFP- or FLAG-GST-tag, along with FLAG-DDX10; cell lysates were subjected to immunoprecipitation and immunoblotting using the indicated antibodies. (**E**) The NLSs within the capsid protein of PCV1, PCV2, PCV3, and PCV4 were responsible for binding to DDX10. HEK293T cells were co-transfected with plasmids encoding NLSs of PCV1, PCV2, PCV3, and PCV4, along with FLAG-DDX10; cell lysates were subjected to immunoprecipitation and immunoblotting using the indicated antibodies. (**F**) The circovirus Cap NLSs from other species were required for interaction with DDX10. HEK293T cells were co-transfected with PCV3-Cap-mNLS and plasmids encoding circovirus Cap NLSs from pig, canary, canine, mink, dragonfly, pigeon, duck, bat, goose, and parrot, along with FLAG-DDX10; cell lysates were subjected to immunoprecipitation and immunoblotting using the indicated antibodies. (**G**) The alignment of the key amino acid residues of circovirus Cap NLS sequences from different species and the mutated PCV3 Cap NLS sequence.

We continued to generate two truncated PCV3 Cap constructs to identify the PCV3 Cap-binding domain responsible for its interaction with DDX10 (Fig. 4A). Coimmunoprecipitation (co-IP) assays revealed that PCV3 Cap or Cap-NLS interacted with DDX10, whereas PCV3 Cap-ΔNLS did not (Fig. 4B through D). These data showed that the PCV3 Cap NLS is the key domain for binding to DDX10. To investigate whether the NLSs within the capsid proteins of PCV1, PCV2, PCV3, or PCV4 interacted with DDX10, Co-IP experiments were performed, and the results indicated that the NLSs within the PCV1, PCV2, PCV3, or PCV4 Cap proteins were required for binding to DDX10 (Fig. 4E). To further confirm the conserved characteristics of the NLS in the interaction between Cap and DDX10, the Cap NLSs of circoviruses from terrestrial, aquatic, and avian species were shown to be essential for interaction with DDX10 (Fig. 4F), demonstrating that the circovirus Cap NLSs from various species are indispensable for binding to DDX10 and that the interactions between DDX10 and circovirus Cap NLSs are highly conserved in evolution. We then analyzed the Cap NLS sequences and the key amino acid sites in the different circovirus species and the mutated PCV3 Cap NLS sequence, and the results indicated that the Cap NLS sequences and the key amino acid sites were highly conserved during evolution (Fig. 4G).

### DDX10 central helicase domain binding to Cap is crucial for regulating PCV3 replication

**Fig 5:**
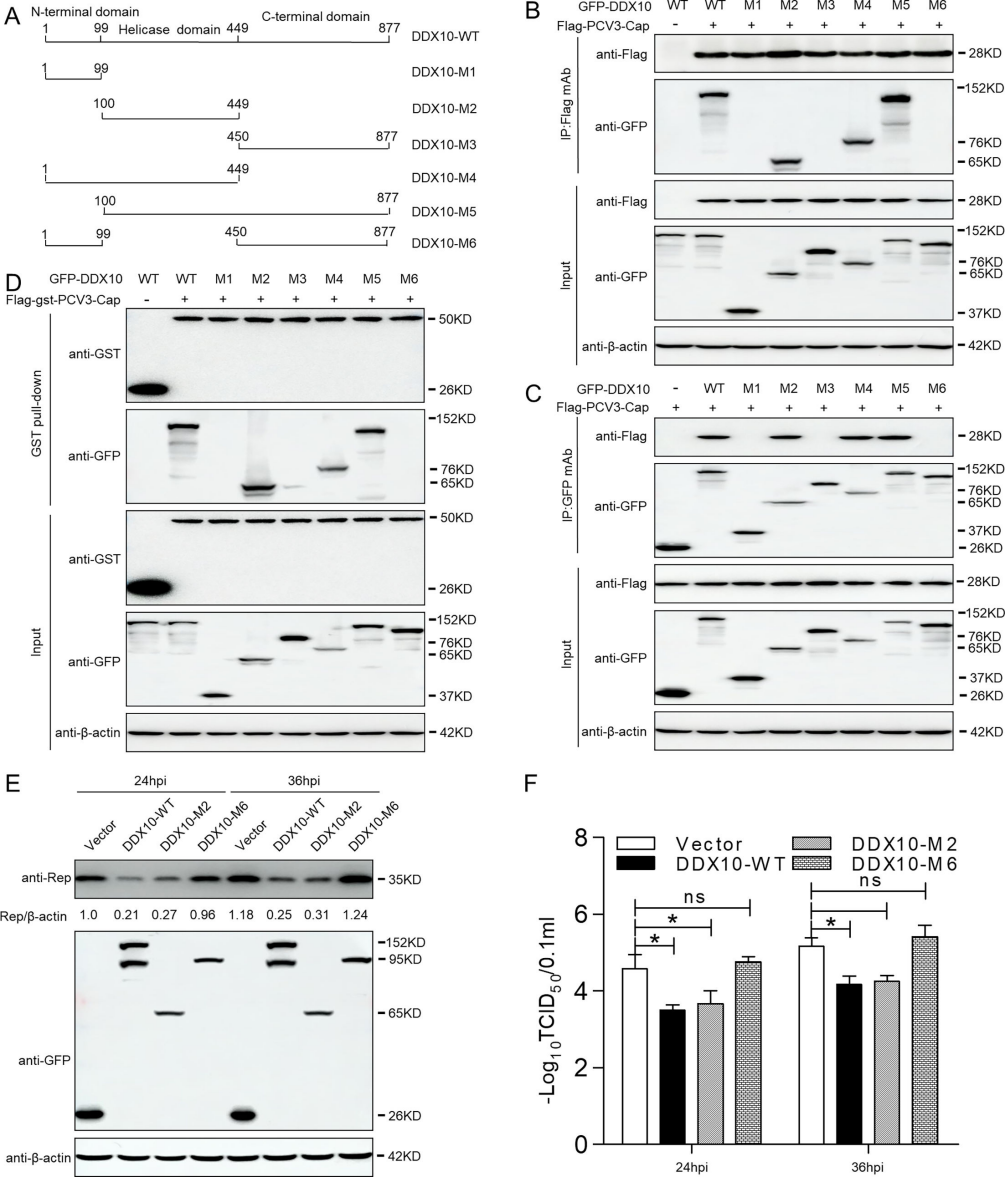
The binding of the central helicase domain of DDX10 binds to Cap, regulating PCV3 replication. (**A**) Schematic representation of the NTD, helicase domain, and CTD of DDX10 and their truncation mutants used in this study. (B–D) The helicase domain of DDX10-(100–449aa) interacted with PCV3 Cap. HEK293T cells were co-transfected with expression plasmids GFP-DDX10-WT or its serial GFP-DDX10 truncated mutants M1–M6, together with Flag-PCV3-Cap or FLAG-GST-PCV3-Cap plasmid. The cell lysate extracts were immunoprecipitated followed by Western blotting using the indicated antibodies. (**E and F**) PK-15 cells were transfected with the indicated GFP-vector, GFP-DDX10-WT, GFP-DDX10-M2-(100–449aa), or GFP-DDX10-M6-(1–99aa + 450–877aa) plasmids for 24 h and were infected with PCV3 at an MOI of 1 for 24 and 36 h. Viral protein and titers were then determined by immunoblotting with anti-GFP, anti-replicase (Rep), and anti-β-actin antibodies and TCID_50_, respectively. The protein intensity band ratio of Rep to β-actin was normalized to control conditions in panels. Data are presented as means ± SD of three independent biological experiments. ns, not significant. *P < 0.05. Figure S1 shows the alignment of amino acid residues of the helicase domain of DDX10 from different genera.

DDX10 has an N-terminal domain, a central helicase, and a C-terminal domain (27). To identify the domain required for DDX10 binding to PCV3 Cap, seven DDX10 truncation mutants fused with GFP, namely, WT-(1–877aa), NTD-M1-(1–99aa), helicase-M2-(100–449aa), CTD-M3-(450–877aa), NTD-helicase-M4-(1–449aa), helicase-CTD-M5-(100–877aa), and NTD-CTD-M6-(1–99aa + 450–877aa), were constructed and mapped to the DDX10 domains required for binding to PCV3 Cap (Fig. 5A). Co-IP assays demonstrated that the DDX10-helicase, DDX10-NTD-helicase, and DDX10-helicase-CTD constructs interacted with PCV3 Cap, whereas the DDX10-NTD, DDX10-CTD, and DDX10-NTD-CTD did not (Fig. 5B through D), indicating that the helicase domain of DDX10 is crucial for binding to PCV3 Cap.

We assessed whether the binding of the central helicase domain of DDX10 to Cap is crucial for modulating PCV3 replication. PK-15 cells were transfected with plasmids expressing GFP-vector, GFP-DDX10-WT, GFP-DDX10-M2-(100–449aa), or GFP-DDX10-M6-(1–99aa + 450–877aa) variants and infected with PCV3 at an MOI of 1 for 24 and 36 h. The Rep protein levels in cells transfected with DDX10-WT or DDX10-M2 were significantly lower than those in cells transfected with the empty vector or DDX10-M6 (Fig. 5E). In addition, the viral titers in DDX10-WT- or DDX10-M2-transfected cells were also significantly decreased at the indicated times (Fig. 5F, *P* < 0.05). These results indicate that DDX10 central helicase domain binding to Cap is essential for PCV3 replication. We then aligned the sequences of the helicase domain of DDX10 from different species via the JALVIEW software suite (version 2.10.5), and the results indicated that the central helicase domain of DDX10 was highly conserved during evolution (Fig. S1). These data demonstrate that the binding of the DDX10 central helicase domain to Cap is crucial for regulating PCV3 replication.

### DDX10 suppresses PCV3 replication by promoting IFN-β production and ISG expression

**Fig 6:**
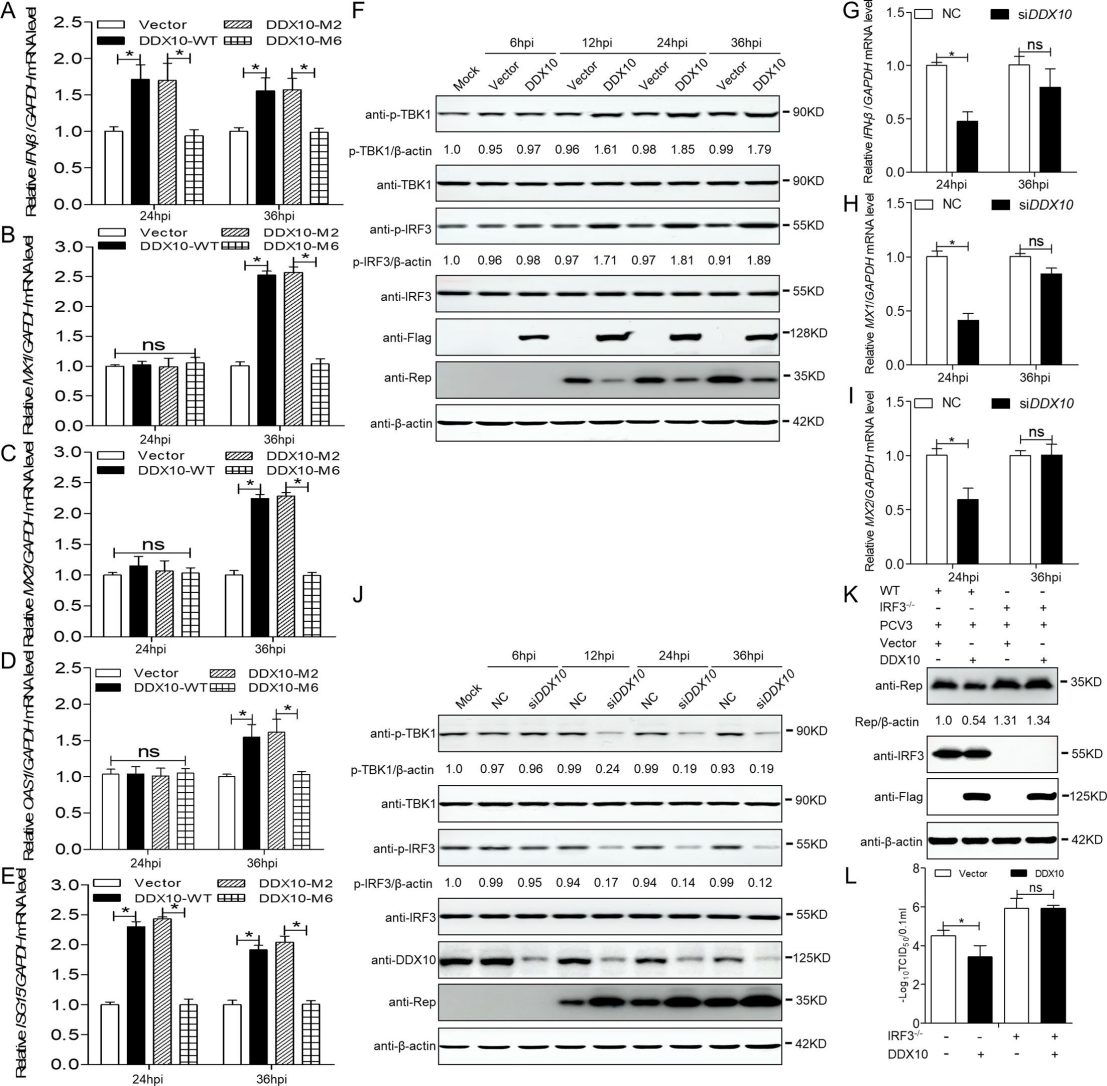
DDX10 increases IFN-β production and ISGs expression to inhibit PCV3 replication. (A–E) RT-qPCR was used to measure the relative expression folds of *IFN-β*, *MX1*, *MX2*, *OAS1*, and *ISG15* normalized to *GAPDH* in GFP-vector, GFP-DDX10-WT, GFP-DDX10-M2-(100–449aa) or GFP-DDX10-M6-(1–99aa + 450–877aa)-overexpressing 3D4/21 cells infected with PCV3 at an MOI of 1 for 24 and 36 h. (**F**) Immunoblotting of the proteins p-TBK1, TBK1, p-IRF3, IRF3, Rep, FLAG, and β-actin in DDX10-overexpressing 3D4/21 cells infected with PCV3 at an MOI of 1 for 6, 12, 24, and 36 h. (G–I) RT-qPCR was used to measure the relative expression folds of *IFN-β*, *MX1*, and *MX2* normalized to *GAPDH* in *DDX10*-silenced 3D4/21 cells infected with PCV3 at an MOI of 1 for the indicated time. (**J**) Immunoblotting of the proteins p-TBK1, TBK1, p-IRF3, IRF3, Rep, DDX10, and β-actin in *DDX10*-silenced 3D4/21 cells infected with PCV3 at an MOI of 1 for 6, 12, 24, and 36 h. (**K and L**) The IRF3^−/−^−3D4/21 cells transfected with FLAG-vector or FLAG-DDX10 were inoculated with PCV3 at an MOI of 1 for 24 h, followed by determination of the Rep, IRF3, FLAG, and β-actin protein levels and viral titers. The protein intensity band ratio of p-TBK1 or p-IRF3 to β-actin was normalized to control conditions in panels **F and J**, respectively. Data are presented as mean ± SD from three independent biological experiments. ns, not significant. *P < 0.05.

DDXs have antiviral activity that primarily regulates IFN-β production or interacts with viral proteins (27, 30, 38, 44). Thus, the effect of porcine *DDX10* on IFN-β production was used to investigate the underlying antiviral mechanism of *DDX10* in PCV3-infected 3D4/21 cells at 24 and 36 hpi. 3D4/21 cells were transfected with the GFP-vector, GFP-DDX10-WT, GFP-DDX10-M2, or GFP-DDX10-M6 for 24 h and then inoculated with PCV3 at an MOI of 1, followed by detection of the mRNA of porcine *IFN-β*. DDX10-WT or DDX10-helicase-M2 overexpression significantly upregulated the mRNA level of *IFN-β* at 24 and 36 hpi, whereas GFP-vector or DDX10-M6 overexpression did not (Fig. 6A, *P* < 0.05). The antiviral effects of IFN-β are primarily due to the induction of many ISGs; therefore, we further verified the role of DDX10 in modulating ISG induction upon PCV3 infection. DDX10-WT or DDX10-M2 overexpression upregulated *MX1*, *MX2*, and *OAS1* mRNA expression at 36 hpi (Fig. 6B through D, *P* < 0.05) and *ISG15* expression at 24 and 36 hpi (Fig. 6E, *P* < 0.05), and DDX10 overexpression significantly increased the p-TBK1 and p-IRF3 protein expression levels at 12, 24, and 36 hpi (Fig. 6F, *P* < 0.05). *DDX10* knockdown also downregulated the *IFN-β*, *MX1*, and *MX2* mRNA expression levels (Fig. 6G through 6I, *P* < 0.05) and significantly reduced the p-TBK1 and p-IRF3 protein expression levels at 12, 24, and 36 hpi (Fig. 6J, *P* < 0.05). To further demonstrate whether IFN induction by the DDX10 protein is required for its anti-PCV3 activity, IRF3^−/−^3D4/21 cells transfected with the FLAG-vector or FLAG-DDX10 were inoculated with PCV3 at an MOI of 1 for 24 h, followed by determining the Rep protein levels and viral titers. The results revealed that DDX10 overexpression did not significantly downregulate Rep protein levels or viral titers in IRF3^−/−^3D4/21 cells (Fig. 6K and L, *P* > 0.05), implying that IFN-β induction by DDX10 is required for its anti-PCV3 activity. Collectively, these data demonstrated that the anti-PCV3 activity of DDX10 is partially connected with the induction of IFN-β production and ISG expression upon PCV3 infection and that PCV3 Cap, in turn, antagonizes its antiviral activity.

## DISCUSSION

Recently, the interplay between viruses and DDXs has attracted increasing attention, as many DDXs have been identified as critical regulators of innate immunity. However, there is only one report on the relationship between viruses and DDX10 (38). Our previous proteomic data revealed that DDX10 interacts with the PCV3 Cap protein (23). Thus, DDX10 was selected for further studies. In this study, we demonstrated that DDX10 expression inhibited PCV3 replication (Fig. 2) by promoting IFN-β production and ISG expression (Fig. 6), and PCV3 infection reduced DDX10 expression levels to tune its antiviral role (Fig. 1), revealing a novel immune evasion strategy utilized by PCV3 infection.

Several DDXs contribute to antiviral immunity by serving as sensors for viral nucleic acids. Pathogens are sensed by innate immune cells via pattern recognition receptors (PRRs) that recognize pathogen-associated molecular patterns (PAMPs) (45). Viral PAMPs are composed of viral genomic RNA or DNA or replication intermediates (46). Double-stranded RNA (dsRNA) has been recognized as a viral PAMP detected by dsRNA-binding proteins (47). The main PRRs are endosomal toll-like receptors (TLRs), cytoplasmic DNA receptors, and RIG-like helicases (RLHs), which belong to the DExH family of RNA helicases (47). In addition, some DExD/H-box helicases have been demonstrated to sense viral nucleic acids (35, 42, 48–51). The main characteristic of antiviral PRR signaling is the induction of IFN-β or cytokines with potent antiviral activity. The antiviral activities of these compounds are largely mediated by ISGs (52). The activation of the transcription factors IRF3 or IRF7 is essential for the induction of IFN-β, and signaling pathways downstream of antiviral PRRs engage kinases that phosphorylate and activate IRF3/7. TLR3, RLHs, and most DNA receptors use the inhibitors of kappa B kinase (IKK)-related kinases TBK1 and IKKε for IRF3/IRF7 phosphorylation (53–58).

As DDX10 expression inhibited PCV3 proliferation, PCV3 infection downregulated the DDX10 level to antagonize antiviral activity, independent of the cell type (Fig. 1 and 2). Many viruses have been demonstrated to evade cellular immune responses by downregulating the expression of cellular proteins, particularly those that activate the IFN-β signaling axis (59–65). For example, the porcine epidemic diarrhea virus reduces HDAC1 expression to promote virus proliferation via the interaction of the nucleocapsid protein with Sp1 (66). Transactive response DNA-binding protein and far upstream element-binding protein 3 inhibit porcine epidemic diarrhea virus replication by degrading the viral nucleocapsid protein and stimulating the type I IFN signaling pathway (67, 68). The herpes simplex virus 1 tegument protein US11 downmodulates the RLR signaling pathway via direct interaction with RIG-I and MDA-5 (69). The African swine fever virus MGF360-14L inhibits type I IFN signaling by targeting IRF3 (70).

In the present study, the results revealed that DDX10 enhances IFN-β production and ISG expression (Fig. 6), which may be a strategy utilized by DDX10 to reduce PCV3 proliferation. However, further studies are needed to determine whether DDX10 interacts with the cGAS-STING-mediated innate signaling axis and how DDX10 activates IFN-β production. In addition to proving that DDX10 enhances IFN-β production, we determined whether DDX10 binds to the PCV3 Cap protein to decrease virus replication. DDX10 interacts with the PCV3 Cap protein (Fig. 3), and the NLS of PCV3 Cap is crucial for binding to the central helicase domain of DDX10, which is required for regulating virus proliferation (Fig. 4 and 5). However, whether DDX10 uses other unidentified mechanisms to inhibit PCV3 infection requires further investigation.

In conclusion, the results show that DDX10 induces IFN-β production and enhances ISG expression, prohibiting PCV3 replication. The NLS of PCV3 Cap is vital for interaction with the central helicase domain of DDX10, which plays an essential role in regulating viral replication. These data increase our understanding of how PCV3 Cap facilitates viral replication and provide novel insight into the prevention and control of PCV3 infection.

## MATERIALS AND METHODS

### Cells and virus determination

The porcine kidney epithelial cell line PK-15 (CCL-33; American Type Culture Collection [ATCC], Manassas, VA, USA) was maintained in a minimal essential medium (Gibco; Thermo Fisher Scientific, Waltham, MA, USA). Porcine pulmonary alveolar macrophages 3D4/21 (ATCC) were cultured in Roswell Park Memorial Institute (RPMI) 1640 medium (Gibco). HEK293T cells (CRL-3216, ATCC) were cultured in Dulbecco’s modified Eagle’s medium (Gibco). All media were supplemented with 10% fetal bovine serum (FBS) (S711-001S, LONSERA; Shanghai Shuangru Biology Science & Technology Co., Ltd., Shuangru, Shanghai, China). The PCV3 LY strain was propagated in PK-15 cells and stored in our laboratory (9). The PK-15 cells were infected with PCV3 according to the following procedure: in a six-well cell culture plate, the PK-15 cells were grown and infected with PCV3 at an MOI of 0.5 for 1 h after reaching 80% confluence and subsequently cultured in minimum essential medium (Gibco) supplemented with 2% FBS in an incubator with 5% CO_2_ at 37°C. The 50% tissue culture infective dose (TCID_50_) assay was used to determine viral titers. Briefly, 10-fold serial dilutions of the harvested cell supernatants were cultured in PK-15 monolayers in 96-well culture plates. Immunofluorescence was used to detect virus titers, which were expressed as the TCID_50_ per 0.1 mL.

### Antibodies and reagents

Rabbit polyclonal antibodies (pAbs) against GFP (SR48-02) and FLAG (0912–1) and mouse mAbs against GST (M0807-1) were purchased from Huaan Biological Technology (Hangzhou, China). Mouse anti-Myc (05–419) or anti-FLAG (F1804) mAbs were purchased from Sigma-Aldrich (St. Louis, MO, USA). Anti-FLAG affinity resin (A2220) for immunoprecipitation was purchased from Sigma-Aldrich. Anti-GFP (B-2, sc-9996) mouse mAbs for immunoprecipitation were acquired from Santa Cruz Biotechnology (Dallas, TX, USA). Mouse anti-β-actin mAbs were purchased from Sangon Biotechnology (D191048; BBI Life Sciences Corp., Shanghai, China). Rabbit pAb against DDX10 (ab243386, A300-617A) was purchased from Abcam (Cambridge, UK) or Bethyl Laboratories. Rabbit mAbs against p-TBK1 (S172) (ab109272), TBK1 (ab40676), and IRF3 (ab68481) were purchased from Abcam. The rabbit pAb anti-p-IRF3 (S396) (AP0623) was purchased from ABclonal Technology. A pig anti-PCV3 Rep antibody was obtained from our laboratory. NP-40 cell lysis buffer (50 mM Tris [pH 7.4], 150 mM NaCl, and 1% NP-40) was purchased from Beyotime (P0013F; Shanghai, China). Horseradish peroxidase- or fluorescein isothiocyanate (FITC)-labeled goat anti-mouse and anti-rabbit IgGs were purchased from KPL (Milford, MA, USA). Rabbit anti-pig IgG was purchased from Bioss (bs-0309Rs; Beijing, China). Alexa Fluor 546-conjugated anti-mouse or anti-rabbit IgG antibodies (Thermo Fisher Scientific) were obtained from Invitrogen Corp. (Carlsbad, CA, USA).

### Plasmid construction and RNA interference

Full-length or truncated PCV3 *Cap* plasmids and NLSs within the capsid proteins of circoviruses from various species (pigs, canaries, canines, minks, dragonflies, pigeons, ducks, bats, geese, and parrots) were constructed as previously described (16). The nucleotide fragment *PCV3 Cap-mNLS* with the positively charged NLS-like amino acid residues Arg, Lys, and His substituted for Ala was synthesized and cloned and inserted into the vector pEGFP-C3 (Clontech Laboratories) by Sangon Biotechnology (Shanghai, China). The full-length cDNA sequences of *NCL* (accession no. XM_021074959.1) and *DDX10* (accession no. XM_021062753.1), and the truncated *DDX10* variants were amplified from PK-15 cells and subsequently cloned and inserted into pCMV-FLAG-N (Clontech Laboratories, Mountain View, CA, USA), pEGFP-C3, pmCherry-C1 (Clontech Laboratories), or pGEX-4T-1 (GE Healthcare Biosciences, Piscataway, NJ, USA) vectors via specific primers. The primers used for cloning and quantitative real-time PCR are listed in Table 1. The resulting plasmids were mCherry-NCL, FLAG-DDX10, GST-DDX10, GFP-DDX10-WT-(1–877aa), GFP-DDX10-M1-(1–99aa), GFP-DDX10-M2-(100–449aa), GFP-DDX10-M3-(450–877aa), GFP-DDX10-M4-(1–449aa), GFP-DDX10-M5-(100–877aa), and GFP-DDX10-M6-(1–99aa +450–877aa). Mutants were generated via site-specific mutagenesis. The nucleotide fragment *Sumo-PCV3-Cap* was synthesized, cloned, and inserted into the vector pET-28a (Invitrogen Corp.) by Sangon Biotechnology. All the constructs were confirmed through sequencing. The cells were seeded in a six-well cell culture plate, grown to 80% confluence, and transfected with Lipofectamine 2000 transfection reagent (11668027, Invitrogen Corp.) for PK-15 cells and ExFect transfection reagent (T101-01/02; Vazyme Biotechnology, Nanjing, China) for HEK293T cells to transfect these recombinant plasmids into PK-15 and HEK293T cells. The GengPharma Company (Suzhou, China) designed siRNAs targeting *DDX10* with the following sequence: si*DDX10* (5′-GCCCAGUACCGCUUGGUAATT-3′). PK-15 cells were transfected with 20 nM siRNA via Lipofectamine RNAiMAX Transfection Reagent (Invitrogen Corp., 13778150) according to the manufacturer’s instructions. Plasmid construction and cell transfection assays were performed as described previously (18).

**TABLE 1:** Primers used for cloning and quantitative real-time PCR

Gene product	Sense primer (5′–3′)	Anti-sense primer (5′–3′)
DDX10-WT-(1–877aa)	ATGGGCAAAACTGCCGATT	CTAGCTTTGATTTTTGAGCAGAT
DDX10-M1-(1–99aa)	ATGGGCAAAACTGCCGATT	TTAGGTCTGCTTCTGGATCTCGGTTA
DDX10-M2-(100–449aa)	ATGATTGGGTTGGCGTTACAAGGTAAAGAT	TTATATAAGTTTTTCTGGATTGATTTT
DDX10-M3-(450–877aa)	ATGGATGTTCAGAAAAAATTGGAATCGTTTTT	CTAGCTTTGATTTTTGAGCAGAT
DDX10-M4-(1–449aa)	ATGGGCAAAACTGCCGATT	TTATATAAGTTTTTCTGGATTGATTTT
DDX10-M5-(100–877aa)	ATGATTGGGTTGGCGTTACAAGGTAAAGAT	CTAGCTTTGATTTTTGAGCAGAT
DDX10-M6-(1–99aa + 450–877aa)	ATGGGCAAAACTGCCGATT	TAAAAACGATTCCAATTTTTTCTGAACATCGGTCTGCTTCTGGATCT
	AGATCCAGAAGCAGACCGATGTTCAGAAAAAATTGGAATCGTTTTTA	CTAGCTTTGATTTTTGAGCAGAT
	ATGGGCAAAACTGCCGATT	CTAGCTTTGATTTTTGAGCAGAT
RT-DDX10	TCCTCAGACAGGAGCGATCA	ACAGAGCACATGCTGGAACA
RT-IFN-β	GTTGCCTGGGACTCCTCAAT	ACGGTTTCATTCCAGCCAGT
RT-MX1	GTCATCGGGGACCAGAGTTC	TCCCGGTAACTGACTTTGCC
RT-MX2	GTCATCGGGGACCAGAGTTC	CTCCACTTTGCGGTAGCTGA
RT-OAS1	GGCTGACCCCACCTACAATG	GGGACTGGGCTCTTGTTGTT
RT-ISG15	GGCAATGTGCTTCAGGATGG	CAGACCTCATAGGCGTTGCT
GAPDH	TCGGAGTGAACGGATTTGGC	TGACAAGCTTCCCGTTCTCC

### Sodium dodecyl sulfate-polyacrylamide gel electrophoresis (SDS-PAGE) and immunoblotting

Cells were lysed in lysis buffer after infection or other treatments as described previously for Western blotting (71). SDS-PAGE and Western blotting were performed as previously described (72).

### Confocal microscopy

Confocal microscopy was used to assess the co-localization of proteins. To test DDX10 and PCV3 Cap or NCL co-localization, PK-15 or HEK293T cells were co-transfected with GFP-DDX10 and mCherry-PCV3-Cap or mCherry-NCL, fixed with 4% paraformaldehyde for 20 min, and permeabilized with 0.2% Triton X-100 for 5 min at room temperature. Cellular nuclei were stained with 10 µg/mL 4′,6′-diamidino-2-phenylindole (10236276001; Roche, Basel, Switzerland). The cells were observed under an LSM780 laser scanning confocal microscope (Zeiss, Oberkochen, Germany) to obtain images under a Zeiss LSM880 confocal laser microscope (Carl Zeiss, Jena, Germany). Confocal microscopy was performed as previously described (71).

### Co-IP and GST pull-down

For Co-IP assays, PK-15 cells were infected with PCV3 at an MOI of 1 for 24 h, or HEK293T cells were transfected with the indicated plasmids for 48 h. The cells were subsequently lysed in NP-40 cell lysis buffer containing a protease inhibitor cocktail. After centrifugation at 12,000 × *g* for 10 min, the supernatant was treated with protein A/G plus agarose (sc-2002, Santa Cruz Biotechnology) for 1 h at 4°C to eliminate nonspecific binding to the agarose beads and then immunoprecipitated with anti-PCV3 Cap mAbs, anti-FLAG beads, anti-GFP mAbs, or glutathione agarose beads. The beads were washed three times with NP-40 buffer and then boiled in a protein-loading buffer before SDS-PAGE and subjected to Western blotting with the indicated antibodies. For the GST pull-down assays, His-Sumo-PCV3-Cap, GST, and GST-DDX10 were separately expressed in *Escherichia coli* BL21 cells. His-Sumo-PCV3-Cap was purified using Ni-NTA agarose (30210; QIAGEN, Germany). GST and GST-DDX10 were purified using Pierce glutathione agarose (21516; Thermo Fisher Scientific, Rockford, IL, USA). To prepare the bait proteins, purified GST and GST-DDX10 were immobilized on glutathione agarose beads (16100, Thermo Fisher Scientific), while lysates of His-Sumo-PCV3-Cap were used as the prey protein. A total of 500 ng of His-Sumo-PCV3-Cap was separately added to the GST and GST-DDX10 proteins, incubated overnight at 4°C, and then washed three times with NP-40 lysis buffer and boiled with sample loading buffer. Finally, the samples were subjected to SDS-PAGE and immunoblotted with anti-His and anti-GST mAbs.

### Quantitative real-time reverse transcription PCR

Two-step real-time reverse transcription PCR (RT-PCR) was performed to measure the mRNA levels of *DDX10*, *IFN-β*, *MX1*, *MX2*, *OAS1*, *ISG15*, and *GAPDH* in 3D4/21 cells. The primers used in the experiments are listed in Table 1. RNA was extracted from whole cells via TRIzol reagent, and a Vazyme cDNA Synthesis Kit (Vazyme Biotechnology) was used to generate cDNA. The cDNA isolated from the samples was further examined via a LightCycler real-time PCR detection device (Roche) and an AceQTM Universal SYBR qPCR Master Mix Kit (Vazyme Biotechnology). The 20.0 µL total volume of the PCR mixture comprised 10.0 µL of AceQTM Universal SYBR qPCR Master Mix, 0.5 µL of cDNA, 9.0 µL of DNase/RNase-free H_2_O, and 0.4 µL of forward and reverse primers. Quantitative RT-PCR was performed as described previously (71).

### Statistical analysis

All the results are presented as the means ± standard deviations. Statistical analysis was performed via Student’s *t*-test. Statistical significance was set as not significant with *P* > 0.05; * denotes *P* < 0.05, and ** denotes *P* < 0.01.

## Data Availability

The data that support the findings of this study are available from the corresponding author upon reasonable request.

## SUPPLEMENTAL MATERIAL

Fig. S1 jvi.00576-25-s0001.pdf Alignment of amino acid residues of helicase domain of DDX10 from different genera.

ASM does not own the copyrights to Supplemental Material that may be linked to, or accessed through, an article. The authors have granted ASM a non-exclusive, world-wide license to publish the Supplemental Material files. Please contact the corresponding author directly for reuse.
