# Supplementary material for: DEAD-box RNA helicase 10 inhibits porcine circovirus type 3 replication by interacting with the viral capsid protein and activating interferon responses
Source: J Virol. 2025 May 9;99(6):e00576-25. doi: 10.1128/jvi.00576-25 (PMC12172478; doi:10.1128/jvi.00576-25)
Supplement: Fig. S1 — Alignment of amino acid residues of helicase domain of DDX10 from different genera. [file jvi.00576-25-s0001.pdf]

Fig. S1

	100 … 219
Sus scrofa	IGLALQGKDVLGAAKTGSGKTLAFL**V**PVLEALYRLQWTS**T**DGLGVLIISPTRELAYQTFEVLRKVGKNHDFSAGLIIGGKDLKHEAERINNINILVCTPGRLLQHMDETICFHATNLQML
Mus musculus	IGLALQGKDVLGAAKTGSGKTLAFL**V**PVLEALYRLQWTS**T**DGLGVLIISPTRELAYQTFEVLRKVGKNHDFSAGLIIGGKDLKHEAERINNINILVCTPGRLLQHMDETICFHATNLQML
Bos taurus	IGLALQGKDVLGAAKTGSGKTLAFL**V**PVLEALYRLQWTS**A**DGLGVLIISPTRELAYQTFEVLRKVGKNHDFSAGLIIGGKDLKHEAERINNINILVCTPGRLLQHMDETICFHATNLQML
Bubalus bubalis	IGLALQGKDVLGAAKTGSGKTLAFL**V**PVLEALYRLQWTS**A**DGLGVLIISPTRELAYQTFEVLRKVGKNHDFSAGLIIGGKDLKHEAERINNINILVCTPGRLLQHMDETICFHATNLQML
Equus asinus	IGLALQGKDVLGAAKTGSGKTLAFL**V**PVLEALYRLQWTS**T**DGLGVLIISPTRELAYQTFEVLRKVGKNHDFSAGLIIGGKDLKHEAERINNINILVCTPGRLLQHMDETICFHATNLQML
Equus caballus	IGLALQGKDVLGAAKTGSGKTLAFL**V**PVLEALYRLQWTS**T**DGLGVLIISPTRELAYQTFEVLRKVGKNHDFSAGLIIGGKDLKHEAERINNINILVCTPGRLLQHMDETICFHATNLQML
Felis catus	IGLALQGKDVLGAAKTGSGKTLAFL**V**PVLEALYRLQWTS**T**DGLGVLIISPTRELAYQTFEVLRKVGKNHDFSAGLIIGGKDLKHEAERINNINILVCTPGRLLQHMDETICFHATNLQML
Marmota flaviventris	IGLALQGKDVLGAAKTGSGKTLAFL**V**PVLEALYRLQWTS**T**DGLGVLIISPTRELAYQTFEVLRKVGKNHDFSAGLIIGGKDLKHEAERINNINILVCTPGRLLQHMDETICFHATNLQML
Canis lupus familiaris	IGLALQGKDVLGAAKTGSGKTLAFL**V**PVLEALYRLQWTS**T**DGLGVLIISPTRELAYQTFEVLRKVGKNHDFSAGLIIGGKDLKHEAERINNINILVCTPGRLLQHMDETICFHATNLQML
Cricetulus griseus	IGLALQGKDVLGAAKTGSGKTLAFL**I**PVLEALYRLQWTS**A**DGLGVLIISPTRELAYQTFEVLRKVGKNHDFSAGLIIGGKDLKHEAERINNINILVCTPGRLLQHMDETICFHATNLQML
Ovis aries	IGLALQGKDVLGAAKTGSGKTLAFL**V**PVLEALYRLQWTS**A**DGLGVLIISPTRELAYQTFEVLRKVGKNHDFSAGLIIGGKDLKHEAERINNINILVCTPGRLLQHMDETICFHATNLQML
**Consensus**	IGLALQGKDVLGAAKTGSGKTLAFL**v**PVLEALYRLQWTS**A**DGLGVLIISPTRELAYQTFEVLRKVGKNHDFSAGLIIGGKDLKHEAERINNINILVCTPGRLLQHMDETICFHATNLQML

	220 … 339
Sus scrofa	VLDEADRILDMGFADTMNAIIENLPKKRQTLLFSATQTKSVKDLARLSLK**N**PEY**V**WVHEKAKYSTPATLEQNYI**V**CEL**Q**QKISVL**Y**SFLRSHLKKKSIVFFSSCKEVQYLYRVFCRLRPG
Mus musculus	VLDEADRILDMGFADTMNAIIENLPKKRQTLLFSATQTKSVKDLARLSLK**D**PEY**V**WVHEKAKYSTPATLEQNYI**I**CEL**H**QKISVL**F**SFLRSHLKKKSIVFFSSCKEVQYLYRVFCRLRPG
Bos taurus	VLDEADRILDMGFADTMNAIIENLPKKRQTLLFSATQTKSVKDLARLSLK**N**PEY**V**WVHEKAKYSTPATLEQNYI**V**CEL**Q**QKISVL**Y**SFLRSHLKKKSIVFFSSCKEVQYLYRVFCRLRPG
Bubalus bubalis	VLDEADRILDMGFADTMNAIIENLPKKRQTLLFSATQTKSVKDLARLSLK**N**PEY**V**WVHEKAKYSTPATLEQNYI**V**CEL**Q**QKISVL**Y**SFLRSHLKKKSIVFFSSCKEVQYLYRVFCRLRPG
Equus asinus	VLDEADRILDMGFADTMNAIIENLPKKRQTLLFSATQTKSVKDLARLSLK**N**PEY**V**WVHEKAKYSTPATLEQNYI**V**CEL**Q**QKISVL**Y**SFLRSHLKKKSIVFFSSCKEVQYLYRVFCRLRPG
Equus caballus	VLDEADRILDMGFADTMNAIIENLPKKRQTLLFSATQTKSVKDLARLSLK**N**PEY**V**WVHEKAKYSTPATLEQNYI**V**CEL**Q**QKISVL**Y**SFLRSHLKKKSIVFFSSCKEVQYLYRVFCRLRPG
Felis catus	VLDEADRILDMGFADTMNAIIENLPKKRQTLLFSATQTKSVKDLARLSLK**N**PEY**V**WVHEKAKYSTPATLEQNYI**V**CEL**Q**QKISVL**Y**SFLRSHLKKKSIVFFSSCKEVQYLYRVFCRLRPG
Marmota flaviventris	VLDEADRILDMGFADTMNAIIENLPKKRQTLLFSATQTKSVKDLARLSLK**N**PEY**V**WVHEKAKYSTPATLEQNYI**V**CEL**Q**QKISVL**Y**SFLRSHLKKKSIVFFSSCKEVQYLYRVFCRLRPG
Canis lupus familiaris	VLDEADRILDMGFADTMNAIIENLPKKRQTLLFSATQTKSVKDLARLSLK**N**PEY**I**WVHEKAKYSTPATLEQNYI**V**CEL**Q**QKISVL**Y**SFLRSHLKKKSIVFFSSCKEVQYLYRVFCRLRPG
Cricetulus griseus	VLDEADRILDMGFADTMNAIIENLPKKRQTLLFSATQTKSVKDLARLSLK**D**PEY**I**WVHEKAKYSTPATLEQNYI**V**CEL**H**QKISVL**F**SFLRSHLKKKSIVFFSSCKEVQYLYRVFCRLRPG
Ovis aries	VLDEADRILDMGFADTMNAIIENLPKKRQTLLFSATQTKSVKDLARLSLK**N**PEY**V**WVHEKAKYSTPATLEQNYI**V**CEL**Q**QKISVL**Y**SFLRSHLKKKSIVFFSSCKEVQYLYRVFCRLRPG
**Consensus**	VLDEADRILDMGFADTMNAIIENLPKKRQTLLFSATQTKSVKDLARLSLK**N**PEY**v**WVHEKAKYSTPATLEQNYI**V**CEL**Q**QKISVL**Y**SFLRSHLKKKSIVFFSSCKEVQYLYRVFCRLRPG

	340 … 449
Sus scrofa	ISILALHGRQQQMRRMEVYNEFVRKKAAVLFATDIAARGLDFPAVNWVLQFDCPEDANTYIHRAGRTARYKEDGEALLILLPSEEKGMVQQLLQKKVPVKEIKINPEKLI
Mus musculus	ISILALHGRQQQMRRMEVYNEFVRKRAAVLFATDIAARGLDFPAVNWVLQFDCPEDANTYIHRAGRTARYKEDGEALLILLPSEEQGMVQQLLQKKVPVKEIKINPEKLI
Bos taurus	VSLLALHGRQQQMRRMEVYNEFVRKRAAVLFATDIAARGLDFPAVNWVLQFDCPEDANTYIHRAGRTARYKEDGEALLILLPSEEKGMVQQLLQKKVPVKEIKINPEKLI
Bubalus bubalis	VSLLALHGRQQQMRRMEVYNEFVRKRAAVLFATDIAARGLDFPAVNWVLQFDCPEDANTYIHRAGRTARYKEDGEALLILLPSEEKGMVQQLLQKKVPVKEIKVNPEKLI
Equus asinus	VSILALHGRQQQMRRMEVYNEFVRKRAAVLFATDIAARGLDFPAVNWVLQFDCPEDANTYIHRAGRTARYKEDGEALLILLPSEEKGMVQQLLQKKVPVKEIKINPEKLI
Equus caballus	VSILALHGRQQQMRRMEVYNEFVRKRAAVLFATDIAARGLDFPAVNWVLQFDCPEDANTYIHRAGRTARYKEDGEALLILLPSEEKGMVQQLLQKKVPVKEIKINPEKLI
Felis catus	ISILALHGRQQQMRRMEVYNEFVRKKAAVLFATDIAARGLDFPAVNWVLQFDCPEDANTYIHRAGRTARYKEDGEALLILLPSEEKGMVQQLLQKKVPVKEIRINPEKLI
Marmota flaviventris	ISILALHGRQQQMRRMEVYNEFVRKRAAVLFATDIAARGLDFPAVNWVLQFDCPEDANTYIHRAGRTARYKEDGEALLILLPSEEKGMVQQLLQKKVPVKEIKINPEKLI
Canis lupus familiaris	ISILALHGRQQQMRRMEVYNEFVRKRSAVLFATDIAARGLDFPAVNWVLQFDCPEDANTYIHRAGRTARYKEDGEALLILLPSEEKGMVQQLLQKKVPVKEIKINPEKLI
Cricetulus griseus	ISILALHGRQQQMRRMEVYNEFLRKRAAVLFATDIAARGLDFPAVNWVLQFDCPEDANTYIHRAGRTARYKEDGEALLILLPSEEQGMVQQLLQKKVPVKEIKINPEKLI
Ovis aries	VSLLALHGRQQQMRRMEVYNEFVRKRAAVLFATDIAARGLDFPAVNWVLQFDCPEDANTYIHRAGRTARYKEDGEALLILLPSEEKGMVQQLLQKKVPVKEIKINPEKLI
**Consensus**	VSLLALHGRQQQMRRMEVYNEFVRKRAAVLFATDIAARGLDFPAVNWVLQFDCPEDANTYIHRAGRTARYKEDGEALLILLPSEEKGMVQQLLQKKVPVKEIKINPEKLI

Fig. S1 Alignment of amino acid residues of helicase domain of DDX10 from different genera
